# Building on a decade of progress in water, sanitation and hygiene to control, eliminate and eradicate neglected tropical diseases

**DOI:** 10.1093/trstmh/trab001

**Published:** 2021-01-28

**Authors:** Sophie Boisson, Leah Wohlgemuth, Aya Yajima, Genandrialine Peralta, Nebe Obiageli, Sultani Matendechero, Gilbert Baayenda, Fikre Seife, Helen Hamilton, Claire Chase, Fatoumata B M Barry, Anthony W Solomon, Yael Velleman

**Affiliations:** Department of Environment, Climate Change and Health, World Health Organization, Geneva, Switzerland; Sightsavers and NTD NGO Network WASH Working Group, London, United Kingdom; Division of Programmes for Disease Control, World Health Organization Regional Office for the Western Pacific, Manila, Philippines; Health and the Environment Programme, World Health Organization Regional Office for the Western Pacific, Manila, Philippines; Department of Public Health, Federal Ministry of Health, Abuja, Nigeria; Division of Vector Borne and Neglected Tropical Diseases, Ministry of Health, Nairobi, Kenya; Trachoma Control Programme, Ministry of Health, Kampala, Uganda; Federal Ministry of Health, Addis Ababa, Ethiopia; WaterAid and NTD NGO Network WASH Working Group, London, United Kingdom; World Bank Water Global Practice, Washington DC, United States; World Bank Health, Nutrition and Population (HNP) Global Practice, Washington DC, United States; Department of Control of Neglected Tropical Diseases, World Health Organization, Geneva, Switzerland; SCI Foundation and NTD NGO Network WASH Working Group, London, United Kingdom

## Abstract

Water, sanitation and hygiene (WASH) are essential for the control and elimination of neglected tropical diseases (NTDs). The forthcoming NTD road map ‘Ending the neglect to attain the Sustainable Development Goals: a road map for neglected tropical diseases 2021–2030’ encourages cross-sectoral collaboration and includes cross-cutting targets on WASH. This commentary reflects on collaborative efforts between the NTD and WASH sectors over the past years and encourages strengthened partnerships to support the new road map and achieve the 2030 agenda ambition of leaving no one behind.

The forthcoming neglected tropical diseases (NTD) publication ‘Ending the neglect to attain the Sustainable Development Goals: a road map for neglected tropical diseases 2021–2030’ encourages a shift from disease-specific programming to comprehensive approaches that involve multiple sectors in NTD control and elimination. The road map includes a cross-cutting target on achieving universal access to ‘at least basic water supply, sanitation and hygiene in areas endemic for NTDs by 2030’.^1^ The target aligns with the Sustainable Development Goal (SDG) targets 6.1 and 6.2 on drinking water and sanitation. It calls for strengthened coordination and collaboration with water, sanitation and hygiene (WASH) stakeholders to ensure that services are delivered and sustained in communities that are most affected by NTDs. It also highlights the crucial role of SDG 6 in meeting the health-related SDG targets. This shift towards comprehensive control of NTDs is crucial since NTDs thrive in areas that lack basic essential services like WASH. Today, large inequalities in access to WASH persist—globally, at least 2 billion people rely on contaminated drinking water supplies, 673 million continue to practice open defecation in the absence of adequate sanitation provisions and 3 billion people have no access to handwashing facilities to practice personal hygiene.^2^ Many more suffer from unreliable or unaffordable services, putting them at risk of exposure to disease and its consequences. Moreover, collaboration across sectors is needed to reduce inequalities and achieve the 2030 agenda of leaving no one behind.

The importance of WASH as stand-alone areas or as a package of services for NTD control and elimination may be insufficiently appreciated. Although positioned as one of the five key strategies to combat NTDs within the 2012–2020 road map,^3^ a lack of clear guidance, practical tools, human resources and clear incentives for collaboration at the time led to a strong focus by NTD programmes on vertical strategies such as mass drug administration and treatment of morbidity. Since 2012, the momentum for WASH and NTD collaboration has grown and was further encouraged through the release of a 5-year World Health Organization (WHO) strategy on WASH and NTDs^4^ in 2015. The strategy provided a framework for collaboration, including awareness raising, using joint monitoring tools to target underserved populations, strengthening and sharing of evidence on effective delivery and joint planning, delivery and evaluation of programmes. Since then, encouraging progress has been made.

## Awareness raising

To sustain the momentum for collaboration, ongoing engagement on WASH and NTDs has continued at global, country and subnational levels. Globally the NTD Non-Governmental Organization (NGO) Network (NNN) has consistently included WASH as a key topic within its annual conferences, while NTD-related sessions have taken place at WASH sector meetings such as the Stockholm World Water Week and the Water and Health conference at the University of North Carolina (Chapel Hill, NC, USA). Several peer-reviewed manuscripts^5,6^ have been published and the third in a series of international roundtables on WASH and NTDs since 2012 took place in Addis Ababa, Ethiopia in 2018. At the national level, dedicated technical working groups in Ethiopia, Guinea-Bissau, Kenya, Nigeria, Senegal, the United Republic of Tanzania and Zimbabwe have provided standing forums for collaboration.

## Joint monitoring

Recognizing the absence of joint monitoring frameworks to incentivize intersectoral collaboration, WASH and NTD stakeholders undertook a detailed consultation to harmonize WASH and NTD indicators.^7^ The resulting indicators have been used in Kenya, Malawi, the United Republic of Tanzania and Uganda, paving the way for the ongoing development of more sophisticated ways for capturing and analysing WASH and NTD data for decision making and for embedding joint indicators in routine WASH and health management information systems.

## Strengthening and sharing of evidence

A comprehensive interactive toolkit^8^ has been developed jointly by the WHO and NNN to enable collaboration between NTD and WASH actors and transition from rhetoric to practice. The resources included in the toolkit have been used to varying degrees in >15 countries across sub-Saharan Africa. A guiding document on the design of effective behaviour change interventions is also under development by the NNN WASH Working Group. A significant challenge is that good-quality evidence of the impact of enhancing WASH access on NTDs is limited and difficult to generate. WASH are complex interventions that are highly context specific and rely on many technologies and behaviours that require time for wide adoption. Recent health impact studies on WASH have highlighted the importance of designing innovative locally appropriate WASH interventions and better documenting their implementation.^9^ Recognizing this challenge, work is under way, led by the WHO and NNN, to develop a formal research agenda; its implementation will allow strengthening of the evidence base on the association between WASH and NTDs, as well as on the effectiveness of WASH interventions in reducing NTD prevalence and incidence.

## Joint planning, delivery and evaluation of programmes

Efforts have focused on enhancing the enabling environment for collaboration, with some large-scale NTD programmes, including the Department of International Development SAFE and Queen Elizabeth Diamond Jubilee Trust trachoma programmes, UK Aid's Ascend, US Agency for International Development’s Act to End and Accelerate programmes, all resourcing WASH and NTDs coordination. There is no doubt, however, that additional work is needed; human and financial resources will be required. Several countries have made important strides towards embedding collaboration within their programmatic structure, with Ethiopia and Kenya appointing dedicated coordination staff, and several others, including Nigeria and Uganda, appointing WASH focal points within their NTD programmes. In Uganda, for example, WASH messages have been included in the training package for NTD mass drug administration and the education and water ministries are to participate in NTD planning and implementation at national and subnational levels. In 2018, Ethiopia established a national planning and reporting framework on WASH and NTDs^10^ as well as a district-level WASH and NTDs toolkit^11^ to inform decentralized planning processes. Lao People's Democratic Republic and Cambodia also led the coordinated implementation of mass drug administration, water safety planning and nutrition interventions to eliminate schistosomiasis among highly endemic communities.^12^

In the short term, the WASH–NTD strategy^4^ will be updated to incorporate lessons learned on WASH–NTD collaboration over the past 5 y and to support the new road map and its ambitious targets. As the coronavirus disease 2019 pandemic evolves, adaptation of NTD programmes, including WASH elements, will be important to ensure continued progress against NTDs. Greater emphasis will be placed on engagement with the WASH sector to drive the necessary targeted investment in endemic communities. Such collaboration will not only ensure that the gains made by NTD control programmes are sustained, but will also pave the way to achieve the overall goals of the sustainable development agenda.

## Data Availability

None.
